# Performance of four scoring systems in predicting the need for massive transfusion in Iranian trauma patients

**DOI:** 10.1371/journal.pone.0353398

**Published:** 2026-07-09

**Authors:** Negin Pouroushaninia, Zahra Ramezani, Atefeh Jahangiri, Niyousha Rahimimovaghar, Gerard O'Reilly, Vali Baigi, Vafa Rahimi-Movaghar

**Affiliations:** 1 Sina Trauma and Surgery Research Center, Tehran University of Medical Sciences, Tehran, Iran; 2 Department of Epidemiology and Biostatistics, School of Public Health, Tehran University of Medical Sciences, Tehran, Iran; 3 UNC Eshelman School of Pharmacy, Chapel Hill, North Carolina, United States of America; 4 School of Public Health and Preventive Medicine, Monash University, Melbourne, Victoria, Australia; 5 National Trauma Research Institute, the Alfred, Melbourne, Victoria, Australia; Jazan University College of Applied Medical Science, SAUDI ARABIA

## Abstract

**Background:**

Post-traumatic hemorrhage is a leading cause of death if not promptly identified and treated. This study aimed to compare the performance of four scoring systems in predicting the need for massive transfusion (MT) in trauma patients.

**Methods:**

This retrospective cohort study analyzed data from trauma patients admitted to Sina Hospital in Tehran between July 2021 and December 2024. All trauma patients who received at least one unit of packed red blood cells were included in the study. Patients were evaluated for the need for MT, and four scoring systems were compared between those who did and did not receive MT. The discriminative ability of the Shock Index (SI), Assessment of Blood Consumption (ABC), Revised Assessment of Bleeding and Transfusion (RABT), and Trauma-Associated Severe Hemorrhage (TASH) was evaluated using the concordance index (C-index) and logistic regression models.

**Results:**

A total of 329 trauma patients were included, with a mean age of 49.2 (SD = 20.8) years; 244 (74.2%) were male. MT occurred in 36 patients (10.9%). All four scoring systems were significantly associated with the need for MT (p < 0.001) and showed comparable discriminative performance (C-index range: 0.81–0.84). At predefined cutoff values, sensitivity ranged from 63.9% to 94.4%, specificity from 60.0% to 94.2%, PPV from 22.4% to 59.5%, and NPV from 95.5% to 98.9%. In univariable logistic regression, an ABC score ≥ 2 was significantly associated with MT (OR: 36.9; 95% CI: 15.6–87.3), followed by an RABT score ≥ 2 (OR: 25.5, 95% CI: 11.2–58.1) and a TASH score ≥ 6 (OR: 25.2, 95% CI: 5.9–106.9).

**Conclusion:**

All four scoring systems showed significant associations with the need for MT. These tools may assist clinicians in the early risk stratification of trauma patients; however, given the moderate positive predictive values in this low-prevalence setting, they should be used as adjuncts to clinical judgment rather than as standalone triggers for massive transfusion activation.

## Introduction

Trauma accounts for more than 9% of deaths globally and is one of the leading causes of disability worldwide. Approximately 4.4 million people die each year due to trauma, and nearly 90% of trauma-related deaths occur in underdeveloped or developing countries [1,2]. In Iran, trauma is responsible for an estimated 28% of annual deaths [3]. Many trauma-related deaths can be prevented with proper and early treatment, with estimates suggesting that approximately 20% of trauma deaths can be avoided [4].

One of the most preventable causes of mortality in trauma patients is post-traumatic hemorrhage (exsanguination), which accounts for more than 1.5 million deaths worldwide annually [4–6]. Nearly 91% of deaths among patients with potentially survivable injuries are caused by bleeding, and 20–40% of in-hospital trauma deaths involve massive hemorrhage [4,7,8]. A life-saving treatment for trauma patients with hemorrhagic shock is Massive Transfusion (MT), although its definition varies across studies [9–11]. Evidence suggests that early diagnosis and management of bleeding in hemorrhagic shock remain major challenges. Previous studies have demonstrated that early administration of blood products within 24 hours can reduce the mortality rate [12]. Predictive tools for bleeding control should be simple, quick, and accessible, and several scoring systems have been developed for this purpose [13], with the simplest being the shock index (SI) [14]. Although numerous scoring systems are generally used to assess overall trauma severity [13,15], some others are specific to hemorrhage in trauma patients, such as the Assessment of Blood Consumption (ABC), Revised Assessment of Bleeding and Transfusion (RABT), and Trauma-Associated Severe Hemorrhage (TASH).

Since most studies on MT prediction have been conducted in developed countries, their findings may not be fully generalizable to developing countries such as Iran. Although a recent study in Iran addressed this topic [16], its analytical approach had some limitations. Therefore, the present study aimed to provide a more rigorous and comprehensive comparison of various scoring systems for predicting MT in trauma patients.

## Methods

### Study design and population

This retrospective cohort study was conducted on trauma patients aged over 18 years admitted to Sina Hospital, affiliated with Tehran University of Medical Sciences. All eligible patients hospitalized between July 2021 and December 2024 who received at least one unit of packed red blood cells (pRBCs) were included. The study methodology adhered to the Strengthening the Reporting of Observational Studies in Epidemiology (STROBE) guidelines. Ethical approval was obtained from the Ethics Committee of Tehran University of Medical Sciences (approval code: IR.TUMS.SINAHOSPITAL.REC.1399.090). The ethical approval was not time-restricted and covered the entire study period. Verbal informed consent was obtained from each patient, or from their next of kin if the patient could not be directly interviewed, for participation in the National Trauma Registry of Iran (NTRI). The consent process was conducted in the presence of an independent witness. The witness confirmed that participants were informed about the study objectives and that participation was voluntary.

### Data collection

Data collection followed two approaches. Some information, including age, gender, systolic blood pressure (SBP), heart rate (HR), oxygen saturation (SpO2), Glasgow Coma Scale (GCS), mechanism of injury, pelvic fracture, height of fall, Emergency Medical Services (EMS) transportation, Abbreviated Injury Scale (AIS), and Injury Severity Score (ISS) were extracted from the existing data in the NTRI. Additional data not available in the registry, such as Focused Assessment with Sonography in Trauma (FAST), the number of pRBCs units received by patients, Hemoglobin (Hb), and base excess (BE), were retrieved from patients’ medical records by two general practitioner investigators using the Hospital Information System (HIS). Medical records were accessed for research purposes between July 2021 and December 2024. These investigators were not involved in the clinical management of the included patients and contributed exclusively in their capacity as researchers to data collection.

The completeness and accuracy of the data collected in the NTRI had been previously evaluated [17], confirming that the registry data are valid and reliable for research purposes. In the current study, the reliability of the extracted variables was further assessed by two independent physicians using both intra- and inter-rater agreement measures. Depending on the type of variable, either Cohen’s kappa coefficient or the intraclass correlation coefficient (ICC) was employed to evaluate agreement levels. All agreement statistics exceeded 0.84, indicating excellent reliability.

In this study, MT was defined as the administration of at least three units of pRBCs within the first three hours of admission [11]. Patients were subsequently classified into two groups based on whether they required MT.

### Scoring systems

The SI was calculated by dividing HR by SBP [18,19]. Another widely used scoring system is the ABC score. This system includes four components: penetrating mechanism of injury, SBP ≤ 90 mmHg, HR ≥ 120/min, and positive FAST result. Each component is assigned one point, and the total score can range from 0 to 4. An increase in its value is positively associated with the likelihood of requiring MT [11]. The RABT includes penetrating mechanism of injury, SI ≥ 1, pelvic fracture, and positive FAST, each of which gets one point, and the overall score ranges from 0 to 4. Higher scores are more closely related to the need for MT [20]. The ABC and RABT scores were dichotomized, with scores of ≥2 classified as positive and those <2 as negative.

The TASH score was calculated using seven distinct variables, including gender, SBP, HR, Hb, FAST, BE, and pelvic fracture. Each variable contributes a specific number of points to the total score, with higher scores indicating an increased risk of MT [13].

### Statistical analysis

Quantitative variables were described using mean and standard deviation or median and interquartile range (IQR), as appropriate. The Mann-Whitney U test was used to compare SBP, HR, RR, GCS, and saturation SpO2 between the two groups. The Chi-square test was applied to assess the association between categorical variables and MT.

The discriminative ability of each scoring system was evaluated using the concordance index (C-index), which corresponds to the area under the receiver operating characteristic (ROC) curve. To address potential overfitting and obtain optimism-corrected estimates, we applied bootstrap resampling with 200 iterations. In each iteration, the C-index was computed for both the bootstrap sample and the original dataset. The average optimism across all iterations was then subtracted from the apparent C-index to derive the bias-corrected estimate. Model calibration was assessed by comparing predicted probabilities to observed outcomes. The calibration slope, which evaluates the agreement between predicted and observed risks, was estimated. A slope near one was interpreted as an indicator of good calibration.

Finally, univariable logistic regression models were used to assess the association between the identified cutoff values of each scoring system and MT. All analyses were performed using Stata version 17 (StataCorp, College Station, TX).

## Results

**Fig 1:**
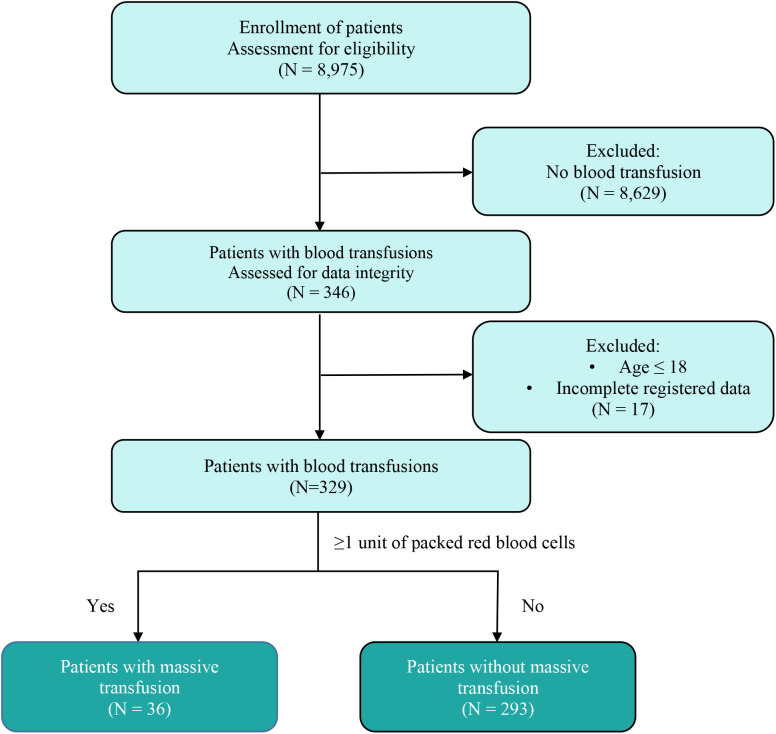
Flowchart of patient selection.

A total of 329 trauma patients were included (Fig 1), with a mean age of 49.2 years (SD = 20.8) (range, 18–91 years). Among all, 244 (74.2%) were male, and 85 (25.8%) were female. MT was required in 36 (10.9%) cases. The mean age of patients receiving MT (36.1 ± 16.8) was significantly lower than that of those not receiving MT (50.8 ± 20.7) (p < 0.001). The need for MT was significantly higher in men than in women (P = 0.003). Patients who received MT had significantly lower SBP and higher HR (p < 0.001). The most prevalent mechanism of injury was blunt trauma, affecting 271 patients (82.4%). Patients with penetrating trauma required MT significantly more than those with blunt trauma (p < 0.001). (Table 1)

**Table 1:** Demographics and injury characteristics of patients stratified by massive transfusion status at admission.

Variable	MT (N = 36)	No MT (N = 293)	p-value
Age (years), mean ± SD	36.1 ± 16.8	50.8 ± 20.7	< 0.001
Sex, N (%)			
Male	34 (13.9)	210 (86.1)	0.003
Female	2 (2.4)	83 (97.6)	
SBP (mmHg), median (IQR)	95.0 (47.0)	120.0 (20.0)	< 0.001
HR (/minutes), median (IQR)	114.0 (32.0)	80.0 (14.0)	< 0.001
SpO2 (%), median (IQR)	98.0 (2.0)	97.0 (2.0)	0.868
GCS, median (IQR)	15.0 (8.0)	15.0 (0.0)	< 0.001
Mechanism of injury, N (%)			
Penetrating	19 (32.7)	39 (67.2)	< 0.001
Blunt	17 (6.3)	254 (93.7)	

MT: Massive Transfusion; SBP: Systolic Blood Pressure; HR: Heart Rate; GCS: Glasgow Coma Score; SD: Standard Deviation; IQR: Interquartile Range; SpO2: Oxygen Saturation

### Scoring systems

A significantly higher median SI was found in patients who received MT (median (IQR) = 1.2 (0.7)) compared to those who did not receive MT (median (IQR) = 0.7 (0.2)) (p < 0.001). Regarding the ABC and RABT scoring systems, the distribution of positive and negative classifications differed significantly between the MT and No MT groups (p < 0.001). Specifically, ABC positivity was observed in 25 patients in the MT group and 17 patients in the No MT group, while RABT positivity was observed in 23 and 19 patients, respectively. Also, the median TASH score differed significantly between the MT and non-MT trauma patients (P < 0.001). (Table 2).

**Table 2:** Comparison of scoring systems between patients with and without massive transfusion.

	MT (N = 36)	No MT (N = 293)	P-value
SI, median (IQR)	1.2 (0.7)	0.7 (0.2)	< 0.001
ABC, n (%)			
Negative	11 (3.8)	276 (96.2)	< 0.001
Positive	25 (59.5)	17 (40.5)	
RABT, n (%)			
Negative	13 (4.5)	274 (95.5)	< 0.001
Positive	23 (54.8)	19 (45.2)	
TASH, median (IQR)	9.5 (3.7)	5.0 (5.5)	< 0.001

MT: Massive Transfusion; SI: Shock Index; ABC: Assessment of Blood Consumption; RABT: Revised Assessment of Bleeding and Transfusion; TASH: Trauma-associated Severe Hemorrhage; IQR: Interquartile Range.

### Predictive performance of the scoring systems for the need for massive transfusion

Table 3 presents the predictive performance details of all four scoring systems for MT. Although there was no statistically significant difference in predictive power based on the C-index, the SI and ABC both demonstrated the highest C-index values (0.84, 95% CI: 0.76 to 0.93).

**Table 3:** Comparative discriminative power of scoring systems for predicting massive transfusion.

	C statistic (95% CI)	Calibration slope (95% CI)
SI	0.84 (0.76 to 0.93)	1.00 (0.66 to 1.44)
ABC	0.84 (0.76 to 0.93)	0.99 (0.76 to 1.33)
RABT	0.81 (0.72 to 0.89)	0.99 (0.72 to 1.30)
TASH	0.84 (0.78 to 0.91)	0.99 (0.69 to 1.36)

I: Shock Index; ABC: Assessment of Blood Consumption; RABT: Revised Assessment of Bleeding and Transfusion; TASH: Trauma-associated severe hemorrhage; AUC: Area under the receiver operating characteristic curve; CI: Confidence interval.

**Fig 2:**
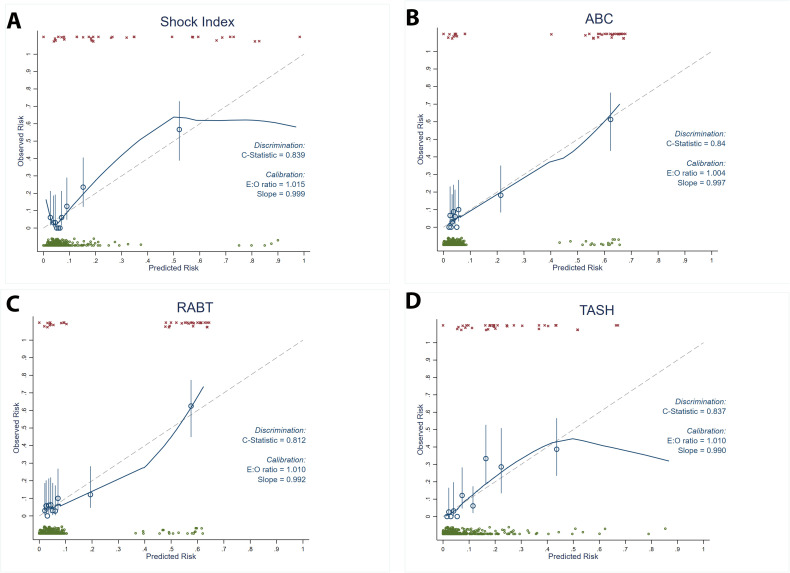
Calibration plots of scoring systems for massive transfusion. C-statistic: Concordance index (C-index), E: O Ratio: Expected-to-observed event ratio.

The calibration plots (Fig 2) suggested generally good agreement between predicted and observed probabilities across all scoring systems, with calibration slopes close to 1. The RABT score showed a calibration slope of 0.99 (95% CI: 0.72 to 1.30).

### Diagnostic performance of the scoring systems

As shown in Table 4, sensitivity ranged from 63.9% (RABT ≥2) to 94.4% (TASH ≥6), whereas specificity ranged from 60.4% (TASH ≥6) to 94.2% (ABC ≥ 2). Positive predictive values (PPV) varied between 22.4% and 59.5%, while negative predictive values (NPV) were consistently high across models (95.5%−98.9%).

**Table 4:** Diagnostic performance of SI, ABC, RABT, and TASH scores for predicting massive transfusion at predefined cutoff values.

	Sen (95% CI)	Spe (95% CI)	PPV (95% CI)	NPV (95% CI)
SI ≥ 0.9	69.4 (51.9 to 83.7)	87.0 (82.6 to 90.7)	40.1 (27.6 to 52.8)	95.9 (92.7 to 97.9)
ABC ≥ 2	69.4 (51.9 to 83.7)	94.2 (90.9 to 96.7)	59.5 (43.3 to 74.4)	96.2 (93.2 to 98.1)
RABT ≥ 2	63.9 (46.2 to 79.2)	93.5 (90.1 to 96.1)	54.8 (38.8 to 70.2)	95.5 (92.4 to 97.6)
TASH ≥ 6	94.4 (81.3 to 99.3)	60.0 (53.9 to 65.4)	22.4 (16.2 to 29.8)	98.9 (96.0 to 99.9)

Sen: Sensitivity, Spe: Specificity, PPV: Positive predictive value, NPV: Negative predictive value

## Logistic regression model

The results of the logistic regression models showed that all scores were significantly associated with MT. Accordingly, the odds of MT in patients with an ABC score ≥ 2 were 36.9 times higher than in patients with an ABC score < 2 (OR: 36.9; 95% CI: 15.6–87.3). (Table 5).

**Table 5:** Univariable logistic regression of determined cutoffs for each scoring system for the massive transfusion.

	OR (95% CI)	p-value
SI ≥ 0.9	15.3 (6.9 to 33.5)	< 0.001
ABC ≥ 2	36.9 (15.6 to 87.3)	< 0.001
RABT ≥ 2	25.5 (11.2 to 58.1)	< 0.001
TASH ≥ 6	25.2 (5.9 to 106.9)	< 0.001

I: Shock Index; ABC: Assessment of Blood Consumption; RABT: Revised Assessment of Bleeding and Transfusion; TASH: Trauma-associated severe hemorrhage; OR: Odds Ratio, CI: Confidence interval.

## Discussion

Uncontrolled hemorrhage after trauma remains a major cause of early preventable death worldwide, and timely recognition of patients at risk for massive transfusion (MT) is a central challenge in trauma resuscitation [13,21–24]. Because delays in activating hemorrhage control strategies and balanced transfusion protocols may worsen outcomes, several bedside scoring systems have been developed to support early identification of patients with severe bleeding [23,25–27]. In the present study, we compared the performance of four commonly used tools—SI, ABC, RABT, and TASH—for predicting MT in a cohort of transfused trauma patients from a major Iranian trauma center. All four scores demonstrated significant associations with MT and showed broadly similar discrimination, with C-index values ranging from 0.81 to 0.84, suggesting that each scoring system has potential value for early hemorrhage risk stratification in this clinical context.

The observed discrimination in our study is consistent with the broader literature showing that SI, ABC, RABT, and TASH can identify trauma patients at increased risk of MT with acceptable to good accuracy [14,16,20,26–29]. Importantly, however, discrimination alone does not determine clinical usefulness. A score with a reasonable C-index may still have limited value if its operating characteristics do not align with the intended clinical purpose. In trauma care, this distinction is particularly relevant because the clinical question is not only whether a patient is statistically at higher risk, but also whether the score can support urgent and actionable decisions during the early phase of resuscitation. For this reason, our interpretation emphasizes not only discrimination but also the balance between sensitivity, specificity, positive predictive value (PPV), and negative predictive value (NPV).

A key finding of our analysis is that although PPV was moderate across models, NPV remained high. This pattern is clinically meaningful and should be interpreted in the context of the relatively low prevalence of MT in our cohort (10.9%). Because predictive values depend on outcome frequency, high NPV in a low-prevalence setting may partly reflect the underlying event rate in addition to score performance [30–32]. The evaluated scores may therefore be more useful for early identification of patients at lower risk for massive transfusion than for definitive identification of patients requiring massive transfusion. Nevertheless, the moderate PPV values indicate that false-positive classifications remain clinically important, and these scores should therefore be interpreted as supportive tools within broader clinical assessment rather than standalone decision rules.

The four scores also differ in their practical implementation requirements, and this has important implications for real-world use. SI is the simplest tool, relying only on heart rate and systolic blood pressure, and may therefore be particularly attractive in prehospital settings, overcrowded emergency departments, and low-resource environments where rapid laboratory testing or sonographic expertise is not always immediately available [14,29]. The ABC score is also relatively practical, integrating mechanism of injury, hemodynamic status, and FAST findings into a simple bedside framework. By contrast, TASH may provide strong discrimination but depends in part on laboratory measures such as hemoglobin and base excess, which may not be available in the earliest moments of trauma evaluation [33]. In this context, the choice among scores may reasonably depend less on small differences in C-index and more on local workflow, timeliness of data acquisition, team familiarity, and the operational structure of trauma resuscitation [34,35]. From a systems perspective, a slightly less complex score that can be applied immediately and consistently may be more useful than a somewhat more detailed model that requires delayed or incomplete data.

Our findings also contribute to the limited evidence from resource-constrained or middle-income trauma settings. In this regard, the study by Safari et al. is particularly relevant, as it evaluated prediction of MT in Iranian trauma patients and reported strong performance for SI and TASH [16]. Their work is important because it highlights that score performance may vary across health systems, case mix, and patterns of injury. However, differences in study design must be considered when comparing results across studies. As noted, their analysis did not include robust internal validation approaches such as bootstrap resampling, which may affect confidence in model stability. In addition, comparing MT recipients with the entire trauma population, rather than with a more clinically similar subgroup of transfused but non-MT patients, may influence apparent predictive performance. Our study instead focused on trauma patients who had already received at least one unit of packed red blood cells, thereby evaluating score performance in a clinically enriched population in whom hemorrhage was sufficiently suspected to prompt transfusion. This design may better reflect the context in which such tools are applied during active resuscitation; however, it also narrows the target population and may reduce generalizability to unselected trauma admissions. Thus, our findings should be interpreted as describing predictive performance in a high-risk, clinically relevant subgroup rather than screening performance across the full trauma spectrum.

An additional methodological issue concerns how these scores were analyzed. In the setting of binary outcomes, the C-index is equivalent to the area under the AUC [36], and thus provides a standard summary of discrimination. However, discrimination should not be conflated with equivalence or interchangeability. Although the scores showed similar C-index values, we did not evaluate whether pairwise differences in AUC were statistically significant, and therefore our findings support broadly comparable rather than definitively identical performance. Likewise, for the ABC and RABT scores, we used their predefined clinical thresholds (≥2), consistent with the original intended use of these tools for bedside decision support and MT activation [26]. While dichotomization may reduce statistical information compared with modeling the full ordinal score [31], preserving established cutoffs enhances clinical interpretability and facilitates comparison with prior validation studies. This is especially relevant for implementation research, where bedside usability and consistency with existing trauma protocols often matter as much as small gains in statistical efficiency.

Overall, our findings support SI, ABC, RABT, and TASH as potentially useful adjunctive tools for early hemorrhage risk stratification in transfused trauma patients, with no clear evidence from this cohort that one score is markedly superior to the others on the basis of discrimination alone. In practice, score selection may be guided by simplicity, immediacy, and data availability rather than by marginal differences in C-index. Nevertheless, caution is warranted before incorporating these tools into protocolized decision pathways as standalone triggers. Future prospective, multicenter, and externally validated studies should assess not only discrimination and calibration, but also clinical utility, including whether implementation of these tools change transfusion timing, protocol activation, blood product use, and patient-centered outcomes. Decision-curve analysis and impact-analysis studies may be especially valuable in determining whether statistical performance translates into meaningful clinical benefit.

### Limitations

Several limitations should be considered. First, because inclusion was restricted to patients who received at least one unit of packed red blood cells, the study cohort may not represent the full clinical spectrum of trauma patients. This selection strategy may have underrepresented clinically important false-positive and false-negative classifications, including patients with occult hemorrhage, patients who died before transfusion initiation, or patients who were never considered for transfusion. Consequently, the observed performance estimates may reflect prediction within a selected clinical-use population rather than true population-level diagnostic performance. Future prospective multicenter studies including broader trauma populations are needed to provide a more comprehensive evaluation of these scoring systems. Second, the relatively low prevalence of MT (10.9%) influenced predictive values, particularly PPV. Because predictive values depend on baseline risk, PPV estimates may differ in trauma populations with higher MT incidence or different case-mix. In addition, although discrimination was good across models, the moderate PPV values observed in this low-prevalence setting indicate that false-positive classifications remain clinically relevant and may limit the utility of these scores as standalone decision tools for massive transfusion activation. Third, the number of MT events (n = 36) was limited. Although discrimination estimates appeared robust, more advanced evaluation of clinical utility, such as decision-curve analysis, may require larger datasets with a greater number of outcome events to provide stable net-benefit estimates across clinically relevant threshold probabilities. Finally, we did not formally assess clinical utility using decision-curve analysis. While the present study focused on discrimination and predictive accuracy, future multicenter or externally validated studies may further evaluate whether implementation of these scores improves clinical decision-making.

## Conclusion

This study showed that SI, ABC, RABT, and TASH had broadly comparable and strong discriminative performance for predicting MT in this cohort of transfused trauma patients. These findings suggest that SI, ABC, RABT, and TASH may provide comparable bedside risk stratification performance in transfused trauma patients. Given the retrospective single-center design and absence of external validation, integration into trauma protocols and generalization to other settings should be approached cautiously and confirmed in prospective multicenter studies. Additionally, future research may evaluate dynamic reassessment approaches and the clinical utility of these tools to determine whether their implementation improves decision-making and patient-centered outcomes.
