# Supplementary material for: Performance of four scoring systems in predicting the need for massive transfusion in Iranian trauma patients
Source: PLoS One. 2026 Jul 9;21(7):e0353398. doi: 10.1371/journal.pone.0353398 (PMC13349299; doi:10.1371/journal.pone.0353398)
Supplement: Attachment — Submitted filename: Response to Reviewer.docx [file pone.0353398.s003.docx]

**Dear Editor,**

We sincerely thank you and the reviewers for the continued time and effort devoted to evaluating our manuscript in this second round of review. We greatly appreciate the constructive and insightful comments, which have further improved the quality and clarity of our work.

We have carefully addressed all comments in a detailed point-by-point response provided below. All corresponding revisions have been incorporated into the manuscript and are clearly highlighted in the tracked-changes version (“Manuscript with Track Changes”). A clean revised version of the manuscript has also been uploaded (“Manuscript”).

We appreciate the opportunity to revise and resubmit our work to *PLOS ONE* and remain available to address any further questions or clarifications

**Journal Requirements:**

1- If the reviewer comments include a recommendation to cite specific previously published works, please review and evaluate these publications to determine whether they are relevant and should be cited. There is no requirement to cite these works unless the editor has indicated otherwise.

**Response:** We have carefully evaluated the reviewer-suggested references and included those deemed relevant and appropriate to the scientific context of the study; citations were not added when they were not directly pertinent.

**Additional Editor Comments:**

2- Please make sure you have read the previous comments made by the reviewers and addressed them accordingly.

**Response:** We thank the Editor for the additional guidance. We have thoroughly reviewed all previous reviewer comments and revised the manuscript accordingly. A detailed point-by-point response is provided in the “Response to Reviewers” document, and all corresponding changes are clearly indicated in the tracked-changes version of the manuscript.

We appreciate the opportunity to further improve our manuscript

**Reviewers' comments:**

**Reviewer #1:** The only meaningful difference from the original manuscript as far as I can see, is the clarification that verbal informed consent was not only obtained but also documented, in response to editorial feedback.

I am unable to identify that additional reviewer comments have been addressed in the manuscript or adequately responded to in the accompanying comments. I therefore further refer to the reviewers’ comments on the original version of the manuscript.

**Response:** Thank you very much for your continued time and careful evaluation of our manuscript.

We apologize if our previous revisions and responses were not sufficiently clear. In the prior revision round, we attempted to address all reviewer comments point by point in the Response to Reviewers document and highlighted all corresponding modifications in the tracked-changes version of the manuscript.

In addition to the clarification regarding documented verbal informed consent requested by the editor, we also revised multiple sections of the manuscript in response to the reviewers’ comments, including modifications to the statistical methodology, interpretation of predictive performance measures, calibration analysis, limitations, and discussion of clinical applicability. These changes were incorporated into both the revised manuscript and the tracked-changes version.

To facilitate reevaluation, we have again provided:

- A detailed point-by-point response to all reviewer comments;
- A revised manuscript with tracked changes highlighting all modifications;
- A clean revised manuscript.

If any comment or concern appears to have been insufficiently addressed, we would be sincerely grateful for further clarification so that we may revise the manuscript accordingly and improve its quality.

We greatly appreciate your valuable feedback and consideration.

**Comment 1:** I am not fully convinced by these conclusions based on the data presented. The authors use the C-index to assess the discriminative ability of each scoring system. This is appropriate for continuous predictors such as the SI and TASH scores. However, the ABC and RABT scores are used as binary outcomes with a cut-off value of 2, and the corresponding numbers are presented in Table 2.

**Response:** We thank the reviewer for this insightful methodological comment. We agree that the interpretation of discrimination measures such as the C-index should be made with consideration of the nature of the predictors, particularly when comparing continuous/ordinal scores with dichotomized variables.

In this study, SI and TASH were analyzed in their continuous/ordinal form, whereas ABC and RABT scores were evaluated using their predefined clinical cut-off values (≥2), consistent with their original derivation and subsequent clinical application for massive transfusion activation in trauma settings[1]. This approach was chosen to reflect real-world bedside decision-making, where these tools are typically applied as rule-based triggers rather than continuous risk scores.

We agree that dichotomization may reduce statistical information and potentially attenuate discrimination compared with modeling the full ordinal score. Nevertheless, the C-index remains a valid measure of discrimination for binary predictors and reflects the ability of the score to separate patients with and without the outcome at the selected threshold[2].

To clarify this issue for readers, we have revised the Discussion section to explicitly acknowledge the potential information loss associated with dichotomization and to emphasize that the use of predefined cutoffs was intended to preserve clinical interpretability and comparability with prior validation studies.

**Changes made in the manuscript:**

***Page 10, Lines 259 to 269 &*** ***Page 11, Lines 270 to 271*** *“An additional methodological issue concerns how these scores were analyzed. In the setting of binary outcomes, the C-index is equivalent to the area under the AUC[3], and thus provides a standard summary of discrimination. However, discrimination should not be conflated with equivalence or interchangeability. Although the scores showed similar C-index values, we did not evaluate whether pairwise differences in AUC were statistically significant, and therefore our findings support broadly comparable rather than definitively identical performance. Likewise, for the ABC and RABT scores, we used their predefined clinical thresholds (≥2), consistent with the original intended use of these tools for bedside decision support and MT activation[1]. While dichotomization may reduce statistical information compared with modeling the full ordinal score[2], preserving established cutoffs enhances clinical interpretability and facilitates comparison with prior validation studies. This is especially relevant for implementation research, where bedside usability and consistency with existing trauma protocols often matter as much as small gains in statistical efficiency.”*

**Comment 2:** Both ABC and RABT yield relatively high C-index values, likely because they perform well as negative predictors of massive transfusion. Yet, when calculating positive predictive value and sensitivity from Table 2, the results are:
• ABC: PPV 59.5%, sensitivity 69.4%, NPV 96.2%, specificity 94.2%
• RABT: PPV 52.3%, sensitivity 63.9%, NPV 95.5%, specificity 92.9%
A PPV of 50–60% may not be sufficient to support the authors’ claim that these scores are useful predictors of massive transfusion. I therefore suggest that predictive values be formally included in the statistical analyses, and that this limitation is adequately discussed in the manuscript.

**Response:** We thank the reviewer for this important comment. In response, we formally incorporated sensitivity, specificity, positive predictive value (PPV), and negative predictive value (NPV), each with 95% confidence intervals, for all scoring systems at their predefined cutoff values (Table 4). We also expanded the Discussion to address the interpretation and clinical implications of the observed predictive values.

In our cohort, the prevalence of massive transfusion was relatively low (10.9%). Accordingly, although ABC and RABT demonstrated high specificity (>93%), PPV values remained moderate (54.8%–59.5%), whereas NPV values were consistently high (>95%). We agree that these findings highlight an important limitation of the evaluated scores and indicate that false-positive classifications remain clinically relevant. We therefore revised the manuscript to emphasize that these tools should be interpreted as supportive risk stratification aids rather than standalone decision rules for massive transfusion activation. We also clarified that predictive values are influenced by outcome prevalence and may vary across trauma populations with different baseline risks.

In accordance with your constructive comments, we have revised the manuscript accordingly. Table 4 presents the diagnostic performance of the SI, ABC, RABT, and TASH scores in predicting massive transfusion at predefined cutoff values.

**Changes made in the manuscript:**

**Page 8, lines 184-188:**

***“Diagnostic performance of the scoring systems***

*As shown in Table 4, sensitivity ranged from 63.9% (RABT ≥2) to 94.4% (TASH ≥6), whereas specificity ranged from 60.4% (TASH ≥6) to 94.2% (ABC ≥2). Positive predictive values (PPV) varied between 22.4% and 59.5%, while negative predictive values (NPV) were consistently high across models (95.5%-98.9%).”*

***“Table 4”***

***Page 9, lines 218-227:*** *“A key finding of our analysis is that although PPV was moderate across models, NPV remained high. This pattern is clinically meaningful and should be interpreted in the context of the relatively low prevalence of MT in our cohort (10.9%). Because predictive values depend on outcome frequency, high NPV in a low-prevalence setting may partly reflect the underlying event rate in addition to score performance[2, 4, 5]. The evaluated scores may therefore be more useful for early identification of patients at lower risk for massive transfusion than for definitive identification of patients requiring massive transfusion. Nevertheless, the moderate PPV values indicate that false-positive classifications remain clinically important, and these scores should therefore be interpreted as supportive tools within broader clinical assessment rather than standalone decision rules.”*

***Page 11, Line 293-299: “****Second, the relatively low prevalence of MT (10.9%) influenced predictive values, particularly PPV. Because predictive values depend on baseline risk, PPV estimates may differ in trauma populations with higher MT incidence or different case-mix. In addition, although discrimination was good across models, the moderate PPV values observed in this low-prevalence setting indicate that false-positive classifications remain clinically relevant and may limit the utility of these scores as standalone decision tools for massive transfusion activation”*

**Comment 3:** In addition, several inconsistencies should be corrected:
• In the abstract, patient inclusion is stated as beginning in July 2022, whereas the Methods section specifies July 1st, 2021.
• The caption of Table 2 notes “comparison of median (IQR),” but medians (IQR) are only provided for two of the four scoring systems; the other two present numbers. The caption should be revised accordingly.
• In Table 2, the total number of patients in the “no MT” group is listed as 293, but summing the RABT values in this group yields 295.

**Answer**: Thank you for identifying these inconsistencies.

- The study inclusion date has been corrected and is now consistent throughout the manuscript.
- The caption of Table 2 has been revised to accurately reflect the data presented.
- The discrepancy in patient numbers has been corrected after rechecking the dataset.

**Page 2, Line 32:** *“between July 2021 and December 2024”*

**Page 6, Lines 163-166:** *“Regarding the ABC and RABT scoring systems, the distribution of positive and negative classifications differed significantly between the MT and No MT groups (p < 0.001). Specifically, ABC positivity was observed in 25 patients in the MT group and 17 patients in the No MT group, while RABT positivity was observed in 23 and 19 patients, respectively.”*

**Reviewer #2**

Reviewer #2: Thank you for the revised manuscript.

While the authors have made some revisions, several of the major concerns raised in the initial review have not been adequately addressed.

In particular, the issues related to selection bias (restriction to transfused patients), overadjustment in the multivariable model, and overinterpretation of similar C-statistics remain insufficiently resolved.
Overall, the revision appears limited, and key methodological concerns persist. I therefore believe the manuscript is not yet suitable for publication in its current form.

**Comment 1 (selection bias (restriction to transfused patients)):** Limiting the study population to patients who received any transfusion restricts the ability to fully evaluate the predictive performance of the scoring systems. Key patient groups that could reveal false positives and false negatives are excluded, including patients who never received transfusion, patients who may have died before transfusion, and those for whom bleeding was underestimated. Discussing the validity and limitations of this approach would help contextualize the interpretation.

**Response:** We sincerely thank the reviewer for this important methodological observation and agree that restricting the cohort to patients who received at least one unit of packed red blood cells may limit the ability to fully evaluate score performance across the entire spectrum of trauma patients. In particular, this design may underrepresent clinically important false-positive and false-negative classifications, including patients who never underwent transfusion evaluation, patients with unrecognized hemorrhage, or patients who died before transfusion could be initiated.

Our intention, however, was to evaluate score performance within a clinically enriched population in whom hemorrhage was suspected and transfusion was actively considered. These tools are primarily intended to support early clinical decision-making in patients perceived to be at risk for major bleeding rather than for universal screening of all trauma admissions. Several prior studies evaluating massive transfusion prediction tools have similarly focused on clinically selected or high-risk trauma populations rather than unselected trauma cohorts[6-10].

Nevertheless, we fully agree that this selection strategy introduces potential spectrum and selection bias and may limit generalizability to unselected trauma populations. We also acknowledge that performance estimates obtained in this selected cohort may differ from true population-level diagnostic performance. To address the reviewer’s concern, we have revised the Discussion and Limitations sections to clarify the intended clinical context of the study population and to explicitly acknowledge the implications of this design on interpretation and generalizability.

**Changes made in the manuscript:**

***Page 10, Lines 251-258:*** *“**Our study instead focused on trauma patients who had already received at least one unit of packed red blood cells, thereby evaluating score performance in a clinically enriched population in whom hemorrhage was sufficiently suspected to prompt transfusion. This design may better reflect the context in which such tools are applied during active resuscitation; however, it also narrows the target population and may reduce generalizability to unselected trauma admissions. Thus, our findings should be interpreted as describing predictive performance in a high-risk, clinically relevant subgroup rather than screening performance across the full trauma spectrum.*

***Page 11, Lines 285-293:*** *“Several limitations should be considered. First, because inclusion was restricted to patients who received at least one unit of packed red blood cells, the study cohort may not represent the full clinical spectrum of trauma patients. This selection strategy may have underrepresented clinically important false-positive and false-negative classifications, including patients with occult hemorrhage, patients who died before transfusion initiation, or patients who were never considered for transfusion. Consequently, the observed performance estimates may reflect prediction within a selected clinical-use population rather than true population-level diagnostic performance. Future prospective multicenter studies including broader trauma populations are needed to provide a more comprehensive evaluation of these scoring systems.”*

**Comment 2:** The multivariable logistic regression raises concerns regarding interpretability and model stability. The model adjusts for variables that are core components of the scoring systems, such as heart rate and systolic blood pressure, resulting in overadjustment that obscures the meaning of the reported associations. In addition, the small number of massive transfusion events relative to the number of covariates increases the risk of overfitting, and the wide confidence intervals indicate substantial imprecision. These adjusted estimates should therefore be interpreted with caution.

**Response:** We sincerely thank the reviewer for this important methodological observation and agree that concerns regarding overadjustment and interpretability of the multivariable regression models remain valid in the context of prediction model evaluation.

Although we previously revised the adjusted models to avoid inclusion of score components as covariates, we recognize that the remaining adjusted analyses may still offer limited interpretability and clinical value. Given the relatively small number of massive transfusion events and the primary predictive focus of the study, we agree that adjusted regression modeling may unnecessarily complicate interpretation and could still raise concerns regarding model stability and overfitting.

In light of the reviewer’s comment, we have therefore removed the multivariable logistic regression analyses from the manuscript and now present the regression results as univariable associations only. We emphasize that the primary evaluation of score performance in this study is based on discrimination, calibration, and diagnostic performance measures, which are more appropriate for validation studies of prediction tools.

The Methods, Results, Abstract, and Table 5 have been revised accordingly.

**Comment 3:** Certain aspects of the interpretation should also be refined to remain consistent with the study design. The C-statistics for all four scores are essentially equivalent, and statements implying superiority based on minor numerical differences should be avoided.

**Response:** We thank the reviewer for this important observation and agree that the C-statistics of the evaluated scoring systems were highly similar and should not be overinterpreted as evidence of meaningful superiority of one score over another.

In response to the reviewer’s concern, we further revised the manuscript to remove or soften language that could imply comparative superiority based on small numerical differences in discrimination measures. We now emphasize throughout the manuscript that SI, ABC, RABT, and TASH demonstrated broadly comparable discriminative performance within this cohort and that the observed differences in C-index values are unlikely to represent clinically meaningful distinctions.

We also clarified that, in the absence of formal statistical superiority testing and given the overlapping confidence intervals, interpretation should focus primarily on the overall comparable predictive performance of these tools rather than ranking individual scores.

The Discussion and Conclusion sections have been revised accordingly.

**References:**

1.	Nunez TC, Voskresensky IV, Dossett LA, Shinall R, Dutton WD, Cotton BA. Early prediction of massive transfusion in trauma: simple as ABC (assessment of blood consumption)? J Trauma. 2009;66(2):346-52. doi: 10.1097/TA.0b013e3181961c35. PubMed PMID: 19204506.

2.	Steyerberg EW. Applications of prediction models. 2 ed: Springer Cham; 2019. XXXIII, 558 p.

3.	Austin PC, Steyerberg EW. Interpreting the concordance statistic of a logistic regression model: relation to the variance and odds ratio of a continuous explanatory variable. BMC Medical Research Methodology. 2012;12(1):82. doi: 10.1186/1471-2288-12-82.

4.	Rothman KJ, Greenland S, Lash TL. Modern epidemiology: Wolters Kluwer Health/Lippincott Williams & Wilkins Philadelphia; 2008.

5.	Szklo M, Nieto FJ. Epidemiology: beyond the basics: Jones & Bartlett Publishers; 2014.

6.	Barbosa Rengifo MM, Garcia AF, Gonzalez-Hada A, Mejia NJ. Evaluating the Shock Index, Revised Assessment of Bleeding and Transfusion (RABT), Assessment of Blood Consumption (ABC) and novel PTTrauma score to predict critical transfusion threshold (CAT) in penetrating thoracic trauma. Scientific reports. 2024;14(1):13395.

7.	Schroll R, Swift D, Tatum D, Couch S, Heaney JB, Llado-Farrulla M, et al. Accuracy of shock index versus ABC score to predict need for massive transfusion in trauma patients. Injury. 2018;49(1):15-9.

8.	Tran A, Matar M, Lampron J, Steyerberg E, Taljaard M, Vaillancourt C. Early identification of patients requiring massive transfusion, embolization or hemostatic surgery for traumatic hemorrhage: a systematic review and meta-analysis. Journal of Trauma and Acute Care Surgery. 2018;84(3):505-16.

9.	Joseph B, Khan M, Truitt M, Jehan F, Kulvatunyou N, Azim A, et al. Massive transfusion: the revised assessment of bleeding and transfusion (RABT) score. World journal of surgery. 2018;42(11):3560-7.

10.	D'Souza K, Norman M, Greene A, Finney CJF, Yan MTS, Trudeau JD, et al. Prediction of massive transfusion with the Revised Assessment of Bleeding and Transfusion (RABT) score at Canadian level I trauma centers. Injury. 2023;54(1):19-24. Epub 20220918. doi: 10.1016/j.injury.2022.09.022. PubMed PMID: 36202645.
