# Supplementary material for: Performance of four scoring systems in predicting the need for massive transfusion in Iranian trauma patients
Source: PLoS One. 2026 Jul 9;21(7):e0353398. doi: 10.1371/journal.pone.0353398 (PMC13349299; doi:10.1371/journal.pone.0353398)
Supplement: Attachment — Submitted filename: Comments.docx [file pone.0353398.s002.docx]

PONE-D-25-45060R1

Performance of Four Scoring Systems in Predicting the Need for Massive Transfusion in Iranian Trauma Patients Dr Vali Baigi

Dear Dr. Baigi,

We've checked your submission and before we can proceed, we need you to address the following issues: 1.

"Thank you for your responses to our previous requests. I apologize that we are requesting further updates, but it is PLOS One's goal to provide the highest standard of reporting in manuscripts that are published with us. We thank you for adjusting your consent statement to include how consent was witnessed. Here we still require how consent was documented. In your previous response, you write that verbal consent was documented in the registry forms. Please add this clarification to the methods section."

Answer:

Thank you for you feedback. We added the clarifying sentence in methods section in page 4. Please let us know if any further amendments are required.
