# Supplementary material for: Performance of four scoring systems in predicting the need for massive transfusion in Iranian trauma patients
Source: PLoS One. 2026 Jul 9;21(7):e0353398. doi: 10.1371/journal.pone.0353398 (PMC13349299; doi:10.1371/journal.pone.0353398)
Supplement: Peer review history

# Peer review: Decision Letter 0

**DOI:** 10.1371/journal.pone.0353398.r001

**Authors:** Rayan Jafnan Alharbi

1 Dec 2025

PONE-D-25-45060

Performance of Four Scoring Systems in Predicting the Need for Massive Transfusion in Iranian Trauma Patients

PLOS ONE

Dear Dr. Baigi,

Thank you for submitting your manuscript to PLOS ONE. After careful consideration, we feel that it has merit but does not fully meet PLOS ONE’s publication criteria as it currently stands. Therefore, we invite you to submit a revised version of the manuscript that addresses the points raised during the review process.

Please submit your revised manuscript by Jan 15 2026 11:59PM. If you will need more time than this to complete your revisions, please reply to this message or contact the journal office at plosone@plos.org. When you’re ready to submit your revision, log on to https://www.editorialmanager.com/pone/ and select the 'Submissions Needing Revision' folder to locate your manuscript file.

Please include the following items when submitting your revised manuscript:

- A rebuttal letter that responds to each point raised by the academic editor and reviewer(s). You should upload this letter as a separate file labeled 'Response to Reviewers'.
- A marked-up copy of your manuscript that highlights changes made to the original version. You should upload this as a separate file labeled 'Revised Manuscript with Track Changes'.
- An unmarked version of your revised paper without tracked changes. You should upload this as a separate file labeled 'Manuscript'.

If you would like to make changes to your financial disclosure, please include your updated statement in your cover letter. Guidelines for resubmitting your figure files are available below the reviewer comments at the end of this letter.

If applicable, we recommend that you deposit your laboratory protocols in protocols.io to enhance the reproducibility of your results. Protocols.io assigns your protocol its own identifier (DOI) so that it can be cited independently in the future. For instructions see: https://journals.plos.org/plosone/s/submission-guidelines#loc-laboratory-protocols. Additionally, PLOS ONE offers an option for publishing peer-reviewed Lab Protocol articles, which describe protocols hosted on protocols.io. Read more information on sharing protocols at https://plos.org/protocols?utm_medium=editorial-email&utm_source=authorletters&utm_campaign=protocols.

We look forward to receiving your revised manuscript.

Kind regards,

Rayan Jafnan Alharbi, Ph.D

Academic Editor

PLOS ONE

Journal Requirements:

When submitting your revision, we need you to address these additional requirements.

1. Please ensure that your manuscript meets PLOS ONE's style requirements, including those for file naming. The PLOS ONE style templates can be found at

https://journals.plos.org/plosone/s/file?id=wjVg/PLOSOne_formatting_sample_main_body.pdf and

https://journals.plos.org/plosone/s/file?id=ba62/PLOSOne_formatting_sample_title_authors_affiliations.pdf

2. In the ethics statement in the Methods, you have specified that verbal consent was obtained. Please provide additional details regarding how this consent was documented and witnessed, and state whether this was approved by the IRB.

3. We note that you have indicated that there are restrictions to data sharing for this study. For studies involving human research participant data or other sensitive data, we encourage authors to share de-identified or anonymized data. However, when data cannot be publicly shared for ethical reasons, we allow authors to make their data sets available upon request. For information on unacceptable data access restrictions, please see http://journals.plos.org/plosone/s/data-availability#loc-unacceptable-data-access-restrictions.

Before we proceed with your manuscript, please address the following prompts:

a) If there are ethical or legal restrictions on sharing a de-identified data set, please explain them in detail (e.g., data contain potentially identifying or sensitive patient information, data are owned by a third-party organization, etc.) and who has imposed them (e.g., a Research Ethics Committee or Institutional Review Board, etc.). Please also provide contact information for a data access committee, ethics committee, or other institutional body to which data requests may be sent.

b) If there are no restrictions, please upload the minimal anonymized data set necessary to replicate your study findings to a stable, public repository and provide us with the relevant URLs, DOIs, or accession numbers. Please see http://www.bmj.com/content/340/bmj.c181.long for guidelines on how to de-identify and prepare clinical data for publication. For a list of recommended repositories, please see https://journals.plos.org/plosone/s/recommended-repositories. You also have the option of uploading the data as Supporting Information files, but we would recommend depositing data directly to a data repository if possible.

Please update your Data Availability statement in the submission form accordingly.

4. We note that your Data Availability Statement is currently as follows:

“All relevant data are within the manuscript and its Supporting Information files.”

Please confirm at this time whether or not your submission contains all raw data required to replicate the results of your study. Authors must share the “minimal data set” for their submission. PLOS defines the minimal data set to consist of the data required to replicate all study findings reported in the article, as well as related metadata and methods (https://journals.plos.org/plosone/s/data-availability#loc-minimal-data-set-definition).

For example, authors should submit the following data:

- The values behind the means, standard deviations and other measures reported;

- The values used to build graphs;

- The points extracted from images for analysis.

Authors do not need to submit their entire data set if only a portion of the data was used in the reported study.

If your submission does not contain these data, please either upload them as Supporting Information files or deposit them to a stable, public repository and provide us with the relevant URLs, DOIs, or accession numbers. For a list of recommended repositories, please see https://journals.plos.org/plosone/s/recommended-repositories.

If there are ethical or legal restrictions on sharing a de-identified data set, please explain them in detail (e.g., data contain potentially sensitive information, data are owned by a third-party organization, etc.) and who has imposed them (e.g., an ethics committee). Please also provide contact information for a data access committee, ethics committee, or other institutional body to which data requests may be sent. If data are owned by a third party, please indicate how others may request data access.

5. If the reviewer comments include a recommendation to cite specific previously published works, please review and evaluate these publications to determine whether they are relevant and should be cited. There is no requirement to cite these works unless the editor has indicated otherwise.

[Note: HTML markup is below. Please do not edit.]

Reviewers' comments:

Reviewer's Responses to Questions

**Comments to the Author**

1. Is the manuscript technically sound, and do the data support the conclusions?

The manuscript must describe a technically sound piece of scientific research with data that supports the conclusions. Experiments must have been conducted rigorously, with appropriate controls, replication, and sample sizes. The conclusions must be drawn appropriately based on the data presented.

Reviewer #1: Partly

Reviewer #2: Partly

**********

2. Has the statistical analysis been performed appropriately and rigorously?

Reviewer #1: No

Reviewer #2: No

**********

3. Have the authors made all data underlying the findings in their manuscript fully available?

The PLOS Data policy requires authors to make all data underlying the findings described in their manuscript fully available without restriction, with rare exception (please refer to the Data Availability Statement in the manuscript PDF file). The data should be provided as part of the manuscript or its supporting information, or deposited to a public repository. For example, in addition to summary statistics, the data points behind means, medians and variance measures should be available. If there are restrictions on publicly sharing data—e.g. participant privacy or use of data from a third party—those must be specified.

Reviewer #1: Yes

Reviewer #2: No

**********

4. Is the manuscript presented in an intelligible fashion and written in standard English?

PLOS ONE does not copyedit accepted manuscripts, so the language in submitted articles must be clear, correct, and unambiguous. Any typographical or grammatical errors should be corrected at revision, so please note any specific errors here.

Reviewer #1: Yes

Reviewer #2: Yes

**********

5. Review Comments to the Author

Please use the space provided to explain your answers to the questions above. You may also include additional comments for the author, including concerns about dual publication, research ethics, or publication ethics. (Please upload your review as an attachment if it exceeds 20,000 characters)

**Reviewer #1:**  Thank you for the opportunity to review this manuscript.

The authors evaluate four different scoring systems for predicting massive transfusion in trauma patients and conclude that all are useful for rapid identification. Furthermore, they state that the SI, ABC, and RABT scores are particularly suited for quick application and should be prioritized in trauma protocols to expedite MT prediction. They recommend immediate integration of these tools into trauma evaluation protocols in Iranian centres, and in other healthcare settings with similar resource constraints.

I am not fully convinced by these conclusions based on the data presented. The authors use the C-index to assess the discriminative ability of each scoring system. This is appropriate for continuous predictors such as the SI and TASH scores. However, the ABC and RABT scores are used as binary outcomes with a cut-off value of 2, and the corresponding numbers are presented in Table 2. Both ABC and RABT yield relatively high C-index values, likely because they perform well as negative predictors of massive transfusion. Yet, when calculating positive predictive value and sensitivity from Table 2, the results are:

• ABC: PPV 59.5%, sensitivity 69.4%, NPV 96.2%, specificity 94.2%

• RABT: PPV 52.3%, sensitivity 63.9%, NPV 95.5%, specificity 92.9%

A PPV of 50–60% may not be sufficient to support the authors’ claim that these scores are useful predictors of massive transfusion. I therefore suggest that predictive values be formally included in the statistical analyses, and that this limitation is adequately discussed in the manuscript.

In addition, several inconsistencies should be corrected:

• In the abstract, patient inclusion is stated as beginning in July 2022, whereas the Methods section specifies July 1st, 2021.

• The caption of Table 2 notes “comparison of median (IQR),” but medians (IQR) are only provided for two of the four scoring systems; the other two present numbers. The caption should be revised accordingly.

• In Table 2, the total number of patients in the “no MT” group is listed as 293, but summing the RABT values in this group yields 295.

**Reviewer #2:**  The authors address the important issue of early identification of patients who may require massive transfusion, and evidence from resource-limited settings is scarce, making data from an Iranian trauma center valuable. Comparing four scoring systems within the same cohort and applying internal validation are notable strengths, and the manuscript is clearly written. At the same time, several methodological aspects require clarification to help readers interpret the findings appropriately.

Limiting the study population to patients who received any transfusion restricts the ability to fully evaluate the predictive performance of the scoring systems. Key patient groups that could reveal false positives and false negatives are excluded, including patients who never received transfusion, patients who may have died before transfusion, and those for whom bleeding was underestimated. Discussing the validity and limitations of this approach would help contextualize the interpretation.

The multivariable logistic regression raises concerns regarding interpretability and model stability. The model adjusts for variables that are core components of the scoring systems, such as heart rate and systolic blood pressure, resulting in overadjustment that obscures the meaning of the reported associations. In addition, the small number of massive transfusion events relative to the number of covariates increases the risk of overfitting, and the wide confidence intervals indicate substantial imprecision. These adjusted estimates should therefore be interpreted with caution.

Certain aspects of the interpretation should also be refined to remain consistent with the study design. The C-statistics for all four scores are essentially equivalent, and statements implying superiority based on minor numerical differences should be avoided. Recommendations to integrate SI or ABC into trauma protocols, or to generalize the findings to other regions, should be stated more cautiously given the retrospective single-center design and absence of external validation. The discussion also links improved predictive accuracy to reductions in preventable mortality, which extends beyond the evidence presented; moderating these statements would improve alignment between results and conclusions.

Minor issues include several repetitions and small inconsistencies in terminology. For example, the “National Trauma Registry of Iran (NTRI)” is defined earlier (L90) and does not need to be reintroduced at L95. Similar minor redundancies and typographical inconsistencies appear elsewhere and would benefit from careful editorial review.

**********

6. PLOS authors have the option to publish the peer review history of their article (what does this mean?). If published, this will include your full peer review and any attached files.

If you choose “no”, your identity will remain anonymous but your review may still be made public.

**Do you want your identity to be public for this peer review?**  For information about this choice, including consent withdrawal, please see our Privacy Policy.

Reviewer #1: No

Reviewer #2: No

**********

[NOTE: If reviewer comments were submitted as an attachment file, they will be attached to this email and accessible via the submission site. Please log into your account, locate the manuscript record, and check for the action link "View Attachments". If this link does not appear, there are no attachment files.]

To ensure your figures meet our technical requirements, please review our figure guidelines: https://journals.plos.org/plosone/s/figures

You may also use PLOS’s free figure tool, NAAS, to help you prepare publication quality figures: https://journals.plos.org/plosone/s/figures#loc-tools-for-figure-preparation.

NAAS will assess whether your figures meet our technical requirements by comparing each figure against our figure specifications.

# Author comment: Author response to Decision Letter 1

**DOI:** 10.1371/journal.pone.0353398.r002

16 Mar 2026

PONE-D-25-45060R1

Performance of Four Scoring Systems in Predicting the Need for Massive Transfusion in Iranian Trauma Patients Dr Vali Baigi

Dear Dr. Baigi,

We've checked your submission and before we can proceed, we need you to address the following issues: 1.

"Thank you for your responses to our previous requests. I apologize that we are requesting further updates, but it is PLOS One's goal to provide the highest standard of reporting in manuscripts that are published with us. We thank you for adjusting your consent statement to include how consent was witnessed. Here we still require how consent was documented. In your previous response, you write that verbal consent was documented in the registry forms. Please add this clarification to the methods section."

Answer:

Thank you for you feedback. We added the clarifying sentence in methods section in page 4. Please let us know if any further amendments are required.

Attachment Submitted filename: Comments.docx

# Peer review: Decision Letter 1

**DOI:** 10.1371/journal.pone.0353398.r003

**Authors:** Rayan Jafnan Alharbi, Rayan Jafnan Alharbi

7 Apr 2026

PONE-D-25-45060R1

Performance of Four Scoring Systems in Predicting the Need for Massive Transfusion in Iranian Trauma Patients

PLOS One

Dear Dr. Baigi,

Thank you for submitting your manuscript to PLOS ONE. After careful consideration, we feel that it has merit but does not fully meet PLOS ONE’s publication criteria as it currently stands. Therefore, we invite you to submit a revised version of the manuscript that addresses the points raised during the review process.

Please submit your revised manuscript by May 22 2026 11:59PM. If you will need more time than this to complete your revisions, please reply to this message or contact the journal office at plosone@plos.org. When you're ready to submit your revision, log on to https://www.editorialmanager.com/pone/ and select the 'Submissions Needing Revision' folder to locate your manuscript file.

Please include the following items when submitting your revised manuscript:

- A letter that responds to each point raised by the academic editor and reviewer(s). You should upload this letter as a separate file labeled 'Response to Reviewers'.
- A marked-up copy of your manuscript that highlights changes made to the original version. You should upload this as a separate file labeled 'Revised Manuscript with Track Changes'.
- An unmarked version of your revised paper without tracked changes. You should upload this as a separate file labeled 'Manuscript'.

If you would like to make changes to your financial disclosure, please include your updated statement in your cover letter. Guidelines for resubmitting your figure files are available below the reviewer comments at the end of this letter.

If applicable, we recommend that you deposit your laboratory protocols in protocols.io to enhance the reproducibility of your results. Protocols.io assigns your protocol its own identifier (DOI) so that it can be cited independently in the future. For instructions see: https://journals.plos.org/plosone/s/submission-guidelines#loc-laboratory-protocols. Additionally, PLOS ONE offers an option for publishing peer-reviewed Lab Protocol articles, which describe protocols hosted on protocols.io. Read more information on sharing protocols at https://plos.org/protocols?utm_medium=editorial-email&utm_source=authorletters&utm_campaign=protocols.

As the corresponding author, your ORCID iD is verified in the submission system and will appear in the published article. PLOS supports the use of ORCID, and we encourage all coauthors to register for an ORCID iD and use it as well. Please encourage your coauthors to verify their ORCID iD within the submission system before final acceptance, as unverified ORCID iDs will not appear in the published article. *Only*  the individual author can complete the verification step; PLOS staff *cannot*  verify ORCID iDs on behalf of authors.

We look forward to receiving your revised manuscript.

Kind regards,

Rayan Jafnan Alharbi, Ph.D

Academic Editor

PLOS One

Journal Requirements:

If the reviewer comments include a recommendation to cite specific previously published works, please review and evaluate these publications to determine whether they are relevant and should be cited. There is no requirement to cite these works unless the editor has indicated otherwise.

Additional Editor Comments:

Please make sure you have read the previous comments made by the reviewers and addressed them accordingly.

[Note: HTML markup is below. Please do not edit.]

Reviewers' comments:

Reviewer's Responses to Questions

**Comments to the Author**

1. If the authors have adequately addressed your comments raised in a previous round of review and you feel that this manuscript is now acceptable for publication, you may indicate that here to bypass the “Comments to the Author” section, enter your conflict of interest statement in the “Confidential to Editor” section, and submit your "Accept" recommendation.

Reviewer #1: (No Response)

Reviewer #2: (No Response)

**********

2. Is the manuscript technically sound, and do the data support the conclusions?

The manuscript must describe a technically sound piece of scientific research with data that supports the conclusions. Experiments must have been conducted rigorously, with appropriate controls, replication, and sample sizes. The conclusions must be drawn appropriately based on the data presented.

Reviewer #1: No

Reviewer #2: Partly

**********

3. Has the statistical analysis been performed appropriately and rigorously?

Reviewer #1: No

Reviewer #2: No

**********

4. Have the authors made all data underlying the findings in their manuscript fully available?

The PLOS Data policy requires authors to make all data underlying the findings described in their manuscript fully available without restriction, with rare exception (please refer to the Data Availability Statement in the manuscript PDF file). The data should be provided as part of the manuscript or its supporting information, or deposited to a public repository. For example, in addition to summary statistics, the data points behind means, medians and variance measures should be available. If there are restrictions on publicly sharing data—e.g. participant privacy or use of data from a third party—those must be specified.

Reviewer #1: No

Reviewer #2: Yes

**********

5. Is the manuscript presented in an intelligible fashion and written in standard English?

PLOS ONE does not copyedit accepted manuscripts, so the language in submitted articles must be clear, correct, and unambiguous. Any typographical or grammatical errors should be corrected at revision, so please note any specific errors here.

Reviewer #1: Yes

Reviewer #2: Yes

**********

6. Review Comments to the Author

Please use the space provided to explain your answers to the questions above. You may also include additional comments for the author, including concerns about dual publication, research ethics, or publication ethics. (Please upload your review as an attachment if it exceeds 20,000 characters)

Reviewer #1: The only meaningful difference from the original manuscript as far as I can see, is the clarification that verbal informed consent was not only obtained but also documented, in response to editorial feedback.

I am unable to identify that additional reviewer comments have been addressed in the manuscript or adequately responded to in the accompanying comments.

I therefore further refer to the reviewers’ comments on the original version of the manuscript.

Reviewer #2: Thank you for the revised manuscript.

While the authors have made some revisions, several of the major concerns raised in the initial review have not been adequately addressed.

In particular, the issues related to selection bias (restriction to transfused patients), overadjustment in the multivariable model, and overinterpretation of similar C-statistics remain insufficiently resolved.

Overall, the revision appears limited, and key methodological concerns persist. I therefore believe the manuscript is not yet suitable for publication in its current form.

**********

7. PLOS authors have the option to publish the peer review history of their article (what does this mean?). If published, this will include your full peer review and any attached files.

If you choose “no”, your identity will remain anonymous but your review may still be made public.

**Do you want your identity to be public for this peer review?**  For information about this choice, including consent withdrawal, please see our Privacy Policy.

Reviewer #1: No

Reviewer #2: No

**********

[NOTE: If reviewer comments were submitted as an attachment file, they will be attached to this email and accessible via the submission site. Please log into your account, locate the manuscript record, and check for the action link "View Attachments". If this link does not appear, there are no attachment files.]

To ensure your figures meet our technical requirements, please review our figure guidelines: https://journals.plos.org/plosone/s/figures

You may also use PLOS’s free figure tool, NAAS, to help you prepare publication quality figures: https://journals.plos.org/plosone/s/figures#loc-tools-for-figure-preparation.

NAAS will assess whether your figures meet our technical requirements by comparing each figure against our figure specifications.

# Author comment: Author response to Decision Letter 2

**DOI:** 10.1371/journal.pone.0353398.r004

19 May 2026

Dear Editor,

We sincerely thank you and the reviewers for the continued time and effort devoted to evaluating our manuscript in this second round of review. We greatly appreciate the constructive and insightful comments, which have further improved the quality and clarity of our work.

We have carefully addressed all comments in a detailed point-by-point response provided below. All corresponding revisions have been incorporated into the manuscript and are clearly highlighted in the tracked-changes version (“Manuscript with Track Changes”). A clean revised version of the manuscript has also been uploaded (“Manuscript”).

We appreciate the opportunity to revise and resubmit our work to PLOS ONE and remain available to address any further questions or clarifications

Journal Requirements:

1- If the reviewer comments include a recommendation to cite specific previously published works, please review and evaluate these publications to determine whether they are relevant and should be cited. There is no requirement to cite these works unless the editor has indicated otherwise.

Response: We have carefully evaluated the reviewer-suggested references and included those deemed relevant and appropriate to the scientific context of the study; citations were not added when they were not directly pertinent.

Additional Editor Comments:

2- Please make sure you have read the previous comments made by the reviewers and addressed them accordingly.

Response: We thank the Editor for the additional guidance. We have thoroughly reviewed all previous reviewer comments and revised the manuscript accordingly. A detailed point-by-point response is provided in the “Response to Reviewers” document, and all corresponding changes are clearly indicated in the tracked-changes version of the manuscript.

We appreciate the opportunity to further improve our manuscript

Reviewers' comments:

Reviewer #1: The only meaningful difference from the original manuscript as far as I can see, is the clarification that verbal informed consent was not only obtained but also documented, in response to editorial feedback.

I am unable to identify that additional reviewer comments have been addressed in the manuscript or adequately responded to in the accompanying comments. I therefore further refer to the reviewers’ comments on the original version of the manuscript.

Response: Thank you very much for your continued time and careful evaluation of our manuscript.

We apologize if our previous revisions and responses were not sufficiently clear. In the prior revision round, we attempted to address all reviewer comments point by point in the Response to Reviewers document and highlighted all corresponding modifications in the tracked-changes version of the manuscript.

In addition to the clarification regarding documented verbal informed consent requested by the editor, we also revised multiple sections of the manuscript in response to the reviewers’ comments, including modifications to the statistical methodology, interpretation of predictive performance measures, calibration analysis, limitations, and discussion of clinical applicability. These changes were incorporated into both the revised manuscript and the tracked-changes version.

To facilitate reevaluation, we have again provided:

- A detailed point-by-point response to all reviewer comments;

- A revised manuscript with tracked changes highlighting all modifications;

- A clean revised manuscript.

If any comment or concern appears to have been insufficiently addressed, we would be sincerely grateful for further clarification so that we may revise the manuscript accordingly and improve its quality.

We greatly appreciate your valuable feedback and consideration.

Comment 1: I am not fully convinced by these conclusions based on the data presented. The authors use the C-index to assess the discriminative ability of each scoring system. This is appropriate for continuous predictors such as the SI and TASH scores. However, the ABC and RABT scores are used as binary outcomes with a cut-off value of 2, and the corresponding numbers are presented in Table 2.

Response: We thank the reviewer for this insightful methodological comment. We agree that the interpretation of discrimination measures such as the C-index should be made with consideration of the nature of the predictors, particularly when comparing continuous/ordinal scores with dichotomized variables.

In this study, SI and TASH were analyzed in their continuous/ordinal form, whereas ABC and RABT scores were evaluated using their predefined clinical cut-off values (≥2), consistent with their original derivation and subsequent clinical application for massive transfusion activation in trauma settings[1]. This approach was chosen to reflect real-world bedside decision-making, where these tools are typically applied as rule-based triggers rather than continuous risk scores.

We agree that dichotomization may reduce statistical information and potentially attenuate discrimination compared with modeling the full ordinal score. Nevertheless, the C-index remains a valid measure of discrimination for binary predictors and reflects the ability of the score to separate patients with and without the outcome at the selected threshold[2].

To clarify this issue for readers, we have revised the Discussion section to explicitly acknowledge the potential information loss associated with dichotomization and to emphasize that the use of predefined cutoffs was intended to preserve clinical interpretability and comparability with prior validation studies.

Changes made in the manuscript:

Page 10, Lines 259 to 269 & Page 11, Lines 270 to 271 “An additional methodological issue concerns how these scores were analyzed. In the setting of binary outcomes, the C-index is equivalent to the area under the AUC[3], and thus provides a standard summary of discrimination. However, discrimination should not be conflated with equivalence or interchangeability. Although the scores showed similar C-index values, we did not evaluate whether pairwise differences in AUC were statistically significant, and therefore our findings support broadly comparable rather than definitively identical performance. Likewise, for the ABC and RABT scores, we used their predefined clinical thresholds (≥2), consistent with the original intended use of these tools for bedside decision support and MT activation[1]. While dichotomization may reduce statistical information compared with modeling the full ordinal score[2], preserving established cutoffs enhances clinical interpretability and facilitates comparison with prior validation studies. This is especially relevant for implementation research, where bedside usability and consistency with existing trauma protocols often matter as much as small gains in statistical efficiency.”

Comment 2: Both ABC and RABT yield relatively high C-index values, likely because they perform well as negative predictors of massive transfusion. Yet, when calculating positive predictive value and sensitivity from Table 2, the results are: • ABC: PPV 59.5%, sensitivity 69.4%, NPV 96.2%, specificity 94.2% • RABT: PPV 52.3%, sensitivity 63.9%, NPV 95.5%, specificity 92.9% A PPV of 50–60% may not be sufficient to support the authors’ claim that these scores are useful predictors of massive transfusion. I therefore suggest that predictive values be formally included in the statistical analyses, and that this limitation is adequately discussed in the manuscript.

Response: We thank the reviewer for this important comment. In response, we formally incorporated sensitivity, specificity, positive predictive value (PPV), and negative predictive value (NPV), each with 95% confidence intervals, for all scoring systems at their predefined cutoff values (Table 4). We also expanded the Discussion to address the interpretation and clinical implications of the observed predictive values.

In our cohort, the prevalence of massive transfusion was relatively low (10.9%). Accordingly, although ABC and RABT demonstrated high specificity (>93%), PPV values remained moderate (54.8%–59.5%), whereas NPV values were consistently high (>95%). We agree that these findings highlight an important limitation of the evaluated scores and indicate that false-positive classifications remain clinically relevant. We therefore revised the manuscript to emphasize that these tools should be interpreted as supportive risk stratification aids rather than standalone decision rules for massive transfusion activation. We also clarified that predictive values are influenced by outcome prevalence and may vary across trauma populations with different baseline risks.

In accordance with your constructive comments, we have revised the manuscript accordingly. Table 4 presents the diagnostic performance of the SI, ABC, RABT, and TASH scores in predicting massive transfusion at predefined cutoff values.

Changes made in the manuscript:

Page 8, lines 184-188:

“Diagnostic performance of the scoring systems

As shown in Table 4, sensitivity ranged from 63.9% (RABT ≥2) to 94.4% (TASH ≥6), whereas specificity ranged from 60.4% (TASH ≥6) to 94.2% (ABC ≥2). Positive predictive values (PPV) varied between 22.4% and 59.5%, while negative predictive values (NPV) were consistently high across models (95.5%-98.9%).”

“Table 4”

Page 9, lines 218-227: “A key finding of our analysis is that although PPV was moderate across models, NPV remained high. This pattern is clinically meaningful and should be interpreted in the context of the relatively low prevalence of MT in our cohort (10.9%). Because predictive values depend on outcome frequency, high NPV in a low-prevalence setting may partly reflect the underlying event rate in addition to score performance[2, 4, 5]. The evaluated scores may therefore be more useful for early identification of patients at lower risk for massive transfusion than for definitive identification of patients requiring massive transfusion. Nevertheless, the moderate PPV values indicate that false-positive classifications remain clinically important, and these scores should therefore be interpreted as supportive tools within broader clinical assessment rather than standalone decision rules.”

Page 11, Line 293-299: “Second, the relatively low prevalence of MT (10.9%) influenced predictive values, particularly PPV. Because predictive values depend on baseline risk, PPV estimates may differ in trauma populations with higher MT incidence or different case-mix. In addition, although discrimination was good across models, the moderate PPV values observed in this low-prevalence setting indicate that false-positive classifications remain clinically relevant and may limit the utility of these scores as standalone decision tools for massive transfusion activation”

Comment 3: In addition, several inconsistencies should be corrected: • In the abstract, patient inclusion is stated as beginning in July 2022, whereas the Methods section specifies July 1st, 2021. • The caption of Table 2 notes “comparison of median (IQR),” but medians (IQR) are only provided for two of the four scoring systems; the other two present numbers. The caption should be revised accordingly. • In Table 2, the total number of patients in the “no MT” group is listed as 293, but summing the RABT values in this group yields 295.

Answer: Thank you for identifying these inconsistencies.

• The study inclusion date has been corrected and is now consistent throughout the manuscript.

• The caption of Table 2 has been revised to accurately reflect the data presented.

• The discrepancy in patient numbers has been corrected after rechecking the dataset.

Page 2, Line 32: “between July 2021 and December 2024”

Page 6, Lines 163-166: “Regarding the ABC and RABT scoring systems, the distribution of positive and negative classifications differed significantly between the MT and No MT groups (p < 0.001). Specifically, ABC positivity was observed in 25 patients in the MT group and 17 patients in the No MT group, while RABT positivity was observed in 23 and 19 patients, respectively.”

Reviewer #2

Reviewer #2: Thank you for the revised manuscript.

While the authors have made some revisions, several of the major concerns raised in the initial review have not been adequately addressed.

In particular, the issues related to selection bias (restriction to transfused patients), overadjustment in the multivariable model, and overinterpretation of similar C-statistics remain insufficiently resolved.

Overall, the revision appears limited, and key methodological concerns persist. I therefore believe the manuscript is not yet suitable for publication in its current form.

Comment 1 (selection bias (restriction to transfused patients)): Limiting the study population to patients who received any transfusion restricts the ability to fully evaluate the predictive performance of the scoring systems. Key patient groups that could reveal false positives and false negatives are excluded, including patients who never received transfusion, patients who may have died before transfusion, and those for whom bleeding was underestimated. Discussing the validity and limitations of this approach would help contextualize the interpretation.

Response: We sincerely thank the reviewer for this important methodological observation and agree that restricting the cohort to patients who received at least one unit of packed red blood cells may limit the ability to fully evaluate score performance across the entire spectrum of trauma patients. In particular, this design may underrepresent clinically important false-positive and false-negative classifications, including patients who never underwent transfusion evaluation, patients with unrecognized hemorrhage, or patients who died before transfusion could be initiated.

Our intention, however, was to evaluate score performance within a clinically enriched population in whom hemorrhage was suspected and transfusion was actively considered. These tools are primarily intended to support early clinical decision-making in patients perceived to be at risk for major bleeding rather than for universal screening of all trauma admissions. Several prior studies evaluating massive transfusion prediction tools have similarly focused on clinically selected or high-risk trauma populations rather than unselected trauma cohorts[6-10].

Nevertheless, we fully agree that this selection strategy introduces potential spectrum and selection bias and may limit generalizability to unselected trauma populations. We also acknowledge that performance estimates obtained in this selected cohort may differ from true population-level diagnostic performance. To address the reviewer’s concern, we have revised the Discussion and Limitations sections to clarify the intended clinical context of the study population and to explicitly acknowledge the implications of this design on interpretation and generalizability.

Changes made in the manuscript:

Page 10, Lines 251-258: “Our study instead focused on trauma patients who had already received at least one unit of packed red blood cells, thereby evaluating score performance in a clinically enriched population in whom hemorrhage was sufficiently suspected to prompt transfusion. This design may better reflect the context in which such tools are applied during active resuscitation; however, it also narrows the target population and may reduce generalizability to unselected trauma admissions. Thus, our findings should be interpreted as describing predictive performance in a high-risk, clinically relevant subgroup rather than screening performance across the full trauma spectrum.

Page 11, Lines 285-293: “Several limitations should be considered. First, because inclusion was restricted to patients who received at least one unit of packed red blood cells, the study cohort may not represent the full clinical spectrum of trauma patients. This selection strategy may have underrepresented clinically important false-positive and false-negative classifications, including patients with occult hemorrhage, patients who died before transfusion initiation, or patients who were never considered for transfusion. Consequently, the observed performance estimates may reflect prediction within a sele

Attachment Submitted filename: Response to Reviewer.docx

# Peer review: Decision Letter 2

**DOI:** 10.1371/journal.pone.0353398.r005

**Authors:** Rayan Jafnan Alharbi, Rayan Jafnan Alharbi, Rayan Jafnan Alharbi

23 Jun 2026

<p>Performance of Four Scoring Systems in Predicting the Need for Massive Transfusion in Iranian Trauma Patients

PONE-D-25-45060R2

Dear Dr. Baigi,

We’re pleased to inform you that your manuscript has been judged scientifically suitable for publication and will be formally accepted for publication once it meets all outstanding technical requirements.

Within one week, you’ll receive an e-mail detailing the required amendments. When these have been addressed, you’ll receive a formal acceptance letter and your manuscript will be scheduled for publication.

An invoice will be generated when your article is formally accepted. Please note, if your institution has a publishing partnership with PLOS and your article meets the relevant criteria, all or part of your publication costs will be covered. Please make sure your user information is up-to-date by logging into Editorial Manager at Editorial Manager® and clicking the ‘Update My Information' link at the top of the page. For questions related to billing, please contact billing support.

If your institution or institutions have a press office, please notify them about your upcoming paper to help maximize its impact. If they’ll be preparing press materials, please inform our press team as soon as possible -- no later than 48 hours after receiving the formal acceptance. Your manuscript will remain under strict press embargo until 2 pm Eastern Time on the date of publication. For more information, please contact onepress@plos.org.

Kind regards,

Rayan Jafnan Alharbi, Ph.D

Academic Editor

PLOS One

Additional Editor Comments (optional):

Reviewers' comments:

Reviewer's Responses to Questions

**Comments to the Author**

1. If the authors have adequately addressed your comments raised in a previous round of review and you feel that this manuscript is now acceptable for publication, you may indicate that here to bypass the “Comments to the Author” section, enter your conflict of interest statement in the “Confidential to Editor” section, and submit your "Accept" recommendation.

Reviewer #1: All comments have been addressed

Reviewer #2: All comments have been addressed

**********

2. Is the manuscript technically sound, and do the data support the conclusions?

The manuscript must describe a technically sound piece of scientific research with data that supports the conclusions. Experiments must have been conducted rigorously, with appropriate controls, replication, and sample sizes. The conclusions must be drawn appropriately based on the data presented.

Reviewer #1: Yes

Reviewer #2: Yes

**********

3. Has the statistical analysis been performed appropriately and rigorously?

Reviewer #1: Yes

Reviewer #2: Yes

**********

4. Have the authors made all data underlying the findings in their manuscript fully available?

The PLOS Data policy requires authors to make all data underlying the findings described in their manuscript fully available without restriction, with rare exception (please refer to the Data Availability Statement in the manuscript PDF file). The data should be provided as part of the manuscript or its supporting information, or deposited to a public repository. For example, in addition to summary statistics, the data points behind means, medians and variance measures should be available. If there are restrictions on publicly sharing data—e.g. participant privacy or use of data from a third party—those must be specified.

Reviewer #1: Yes

Reviewer #2: Yes

**********

5. Is the manuscript presented in an intelligible fashion and written in standard English?

PLOS ONE does not copyedit accepted manuscripts, so the language in submitted articles must be clear, correct, and unambiguous. Any typographical or grammatical errors should be corrected at revision, so please note any specific errors here.

Reviewer #1: Yes

Reviewer #2: Yes

**********

6. Review Comments to the Author

Please use the space provided to explain your answers to the questions above. You may also include additional comments for the author, including concerns about dual publication, research ethics, or publication ethics. (Please upload your review as an attachment if it exceeds 20,000 characters)

Reviewer #1: Thank you for the opportunity to re-review this manuscript. The authors have satisfactorily addressed my concerns, and I consider the revised manuscript suitable for publication.

Reviewer #2: The authors have adequately addressed my previous major comments. I have no further major concerns. I support acceptance of the revised manuscript.

**********

7. PLOS authors have the option to publish the peer review history of their article (what does this mean?). If published, this will include your full peer review and any attached files.

If you choose “no”, your identity will remain anonymous but your review may still be made public.

**Do you want your identity to be public for this peer review?**  For information about this choice, including consent withdrawal, please see our Privacy Policy.

Reviewer #1: No

Reviewer #2: No

**********

# Editor report: Acceptance letter

**DOI:** 10.1371/journal.pone.0353398.r006

**Authors:** Rayan Jafnan Alharbi, Rayan Jafnan Alharbi, Rayan Jafnan Alharbi

PONE-D-25-45060R2

PLOS One

Dear Dr. Baigi,

I'm pleased to inform you that your manuscript has been deemed suitable for publication in PLOS One. Congratulations! Your manuscript is now being handed over to our production team.

At this stage, our production department will prepare your paper for publication. This includes ensuring the following:

* All references, tables, and figures are properly cited

* All relevant supporting information is included in the manuscript submission,

* There are no issues that prevent the paper from being properly typeset

You will receive further instructions from the production team, including instructions on how to review your proof when it is ready. Please keep in mind that we are working through a large volume of accepted articles, so please give us a few days to review your paper and let you know the next and final steps.

Lastly, if your institution or institutions have a press office, please let them know about your upcoming paper now to help maximize its impact. If they'll be preparing press materials, please inform our press team within the next 48 hours. Your manuscript will remain under strict press embargo until 2 pm Eastern Time on the date of publication. For more information, please contact onepress@plos.org.

You will receive an invoice from PLOS for your publication fee after your manuscript has reached the completed accept phase. If you receive an email requesting payment before acceptance or for any other service, this may be a phishing scheme. Learn how to identify phishing emails and protect your accounts at https://explore.plos.org/phishing.

If we can help with anything else, please email us at customercare@plos.org.

Thank you for submitting your work to PLOS ONE and supporting open access.

Kind regards,

PLOS ONE Editorial Office Staff

on behalf of

Dr. Rayan Jafnan Alharbi

Academic Editor

PLOS One
